# Neuroimaging in dementia

**DOI:** 10.1007/s10354-021-00825-x

**Published:** 2021-03-03

**Authors:** Julia Furtner, Daniela Prayer

**Affiliations:** Department of Biomedical Imaging and Image-guided Therapy, Medical University of Vienna, Waehringerguertel 18–20, 1090 Vienna, Austria

**Keywords:** Magnetic resonance imaging, Dementia, Alzheimer’s Disease, Vascular Dementia, Magnetresonanztomografie, Demenz, Alzheimer, Vaskuläre Demenz

## Abstract

Despite the fact that the diagnosis of dementia is mainly based on clinical criteria, the role of neuroimaging is still expanding. Among other imaging techniques, magnetic resonance imaging (MRI) plays a core role in assisting with the differentiation between various dementia syndromes and excluding other underlying pathologies that cause dementia, such as brain tumors and subdural hemorrhages. This article gives an overview of the standard MRI protocol and of structural radiological reporting systems in patients who suffer from dementia. Moreover, it presents characteristic MRI features of the most common dementia subtypes.

## Abstract (de)

Trotz der Tatsache, dass Demenzdiagnostik vor allem auf klinischen Kriterien basiert, erhält die Bildgebung einen immer höheren Stellenwert. Neben diversen weitern bildgebenden Verfahren spielt die Magnetresonanztomographie (MRT) eine zentrale Rolle bei der Differenzierung zwischen den unterschiedlichen Demenzsyndromen bzw. zum Ausschluss etwaiger anderer zugrunde liegender Pathologien, die eine Demenz verursachen können, wie Hirntumoren und subdurale Blutungen. Dieser Artikel gibt einerseits einen Überblick über standardisierte MRT-Protokolle sowie andererseits über eine strukturierte radiologische Befundung im Rahmen einer bildgebenden Demenzabklärung. Darüber hinaus werden charakteristische MRT-Merkmale der häufigsten Demenz-Subtypen vorgestellt.

## Introduction

Neuroimaging plays a major role in the diagnostic work-up of patients who suffer from dementia. Computed tomography (CT) or magnetic resonance imaging (MRI) scans are essential to rule out underlying, treatable causes of cognitive impairment, such as brain tumors, subdural hematomas, or normal pressure hydrocephalus. However, neuroimaging is increasingly included in the diagnostic criteria of diseases that cause dementia.

## MR imaging protocol and standardized radiological reporting

Recommended MRI protocols in patients who suffer from dementia include the following MRI sequences:

- *T2-weighted and Fluid-attenuated recovery (Flair) MR sequences*
  Optional 2D or 3D; to identify signal abnormalities within the gray and white matter including hippocampal signal alterations and to determine the degree of vascular damage comprising white matter hyperintensities, lacunes, and post-ischemic parenchymal defects
- *T1-weighted MR sequences*
  3D isotropic; to assess the pattern and extent of brain atrophy (including global cortical atrophy (GCA), medial temporal atrophy (MTA) scores and the posterior/parietal atrophy score)
- *Diffusion-weighted MR images* (DWI)
  Axial 2D; to display areas with diffusion restriction indicative of acute ischemia, inflammation, or signal alterations with regard to Creutzfeld Jacob disease
- *Susceptibility-weighted MR images* (SWI)
  Axial 2D; to represent microbleeds or superficial siderosis.

The application of a gadolinium-based MR contrast agent is not routinely indicated; however, it may be helpful in atypical cases, such as suspected infection, tumorous lesions, or vasculitis.

The radiological report of patients suspected of having dementia should routinely include a standardized assessment of the following features:

1. *Global cortical atrophy*
  The four-step global cortical atrophy (GCA) scale proposed by Pasquier ranges from 0 = no atrophy to 3 = knife-blade atrophy (illustrated in Fig. 1; [1]). GCA 0 represents a normal volume of the gyri and a normal width of the sulci; GCA 1 indicates mild atrophy with a still-normal volume of the gyri but some open sulci; GCA 2 describes moderate brain atrophy with a reduction of gyri volume and a moderate enlargement of the sulci; and GCA 3 illustrates severe atrophy with severely reduced gyri and enlarged sulci. In addition, the focal regions of brain atrophy should be given.

**Fig. 1:**
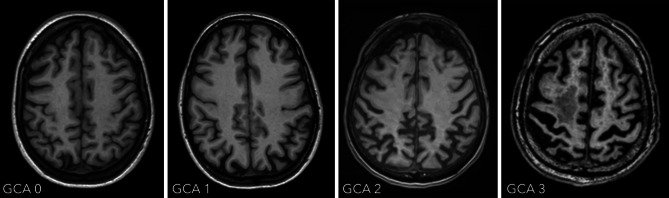
Global cortical atrophy scale
2. *Medial temporal lobe atrophy*
  The degree of medial temporal lobe atrophy can be assessed using the medial temporal lobe atrophy (MTA) score assessed on coronal planes perpendicular to the long axis of the hippocampus (illustrated in Fig. 2). MTA 0 presents a normal width of the choroid fissure, the temporal horn, and a normal hippocampal volume; MTA 1 is characterized by a marginally widened choroid fissure; MTA 2 shows a moderately widened choroid fissure, a mild enlargement of the temporal horn, and a mild loss of hippocampal volume; MTA 3 is characterized by a markedly widened choroid fissure, a moderate enlargement of the temporal horn, and a moderate loss of hippocampal volume; and MTA 4 is defined as a markedly widened choroid fissure, a marked enlargement of the temporal horn, as well as a marked loss of hippocampal volume.

**Fig. 2:**
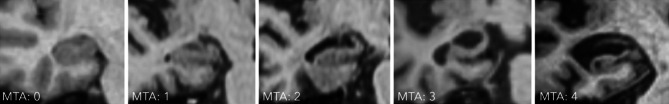
Medial temporal atrophy scale
3. *Posterior/parietal atrophy*
  The posterior/parietal atrophy score (Koedam score) has been designed to visually evaluate the posterior cingulate, precuneus and superior parietal regions on a four-step scale (illustrated in Fig. 3; [2]). To generate the score, the MR images of the brain have to be viewed in axial, coronal and sagittal planes assessing the following structures:
  sagittal plane:
    precuneus gyrus (PRE)
    posterior cingulate sulcus (PCS)
    parieto-occipital sulcus (POS)
  axial plane
    posterior cingulate sulcus (PCS)
    parietal gyrus (PAR)
  coronal plane
    posterior cingulate sulcus (PCS)
    parietal gyrus (PAR)
  A posterior/﻿parietal atrophy score of 0 represents a normal width of the above mentioned sulci and no atrophy of the precuneus, while score of 1, 2 or 3 is characterized by mild, moderate or severe widening of the sulci and precuneus atrophy, respectively.

4. *White matter changes*
  The most commonly used rating scale to determine the amount of white matter changes is the three-step Fazekas scale (illustrated in Fig. 4). It differentiates between small punctate (Fazekas 1), early confluent (Fazekas 2), and marked confluent (Fazekas 3) white matter lesions. For the diagnoses of vascular dementia, the more precise criteria of the National Institute of Neurological Disorders and Stroke and the Association Internationale pour la Recherché et l’Enseignement en Neurosciences (NINSDS-AIREN criteria) are taken into account [3].
5. *Lacunes and Virchow-Robin spaces*
  Lacunes are deep, small-vessel infarcts with a CSF-like signal on all MRI sequences (illustrated in Fig. 5). In contrast, Virchow-Robin spaces are enlarged perivascular spaces usually due to volume loss of the surrounding tissue with a predilection for the basal ganglia (illustrated in Fig. 6).
6. *Microbleeds*
  Microbleeds are small punctate areas of low signal intensity on T2*-weighted images related to hemosiderin deposition in vessel walls with regard to arteriosclerosis (illustrated in Fig. 7).

**Fig. 3:**
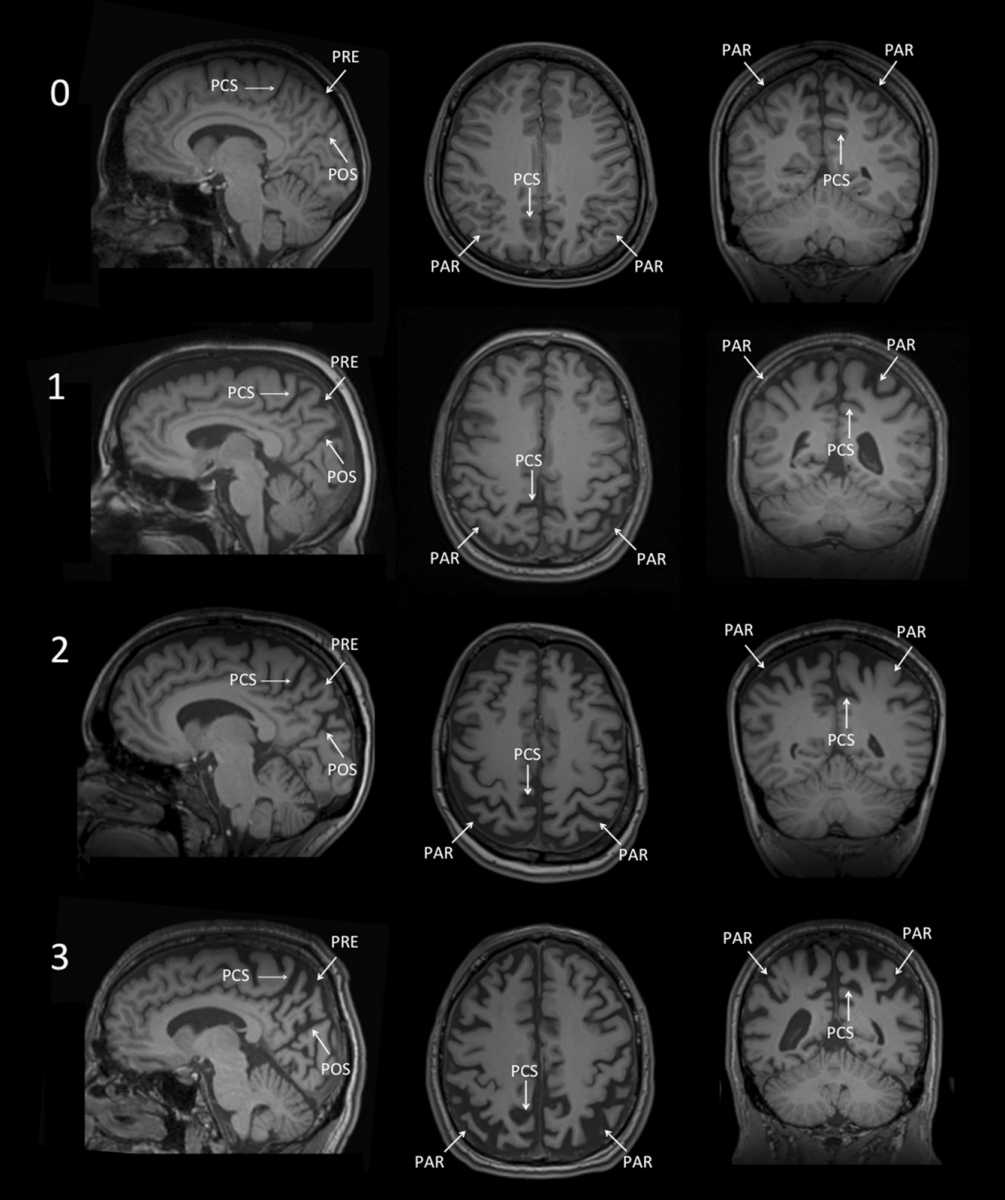
Posterior/parietal atrophy score

**Fig. 4:**
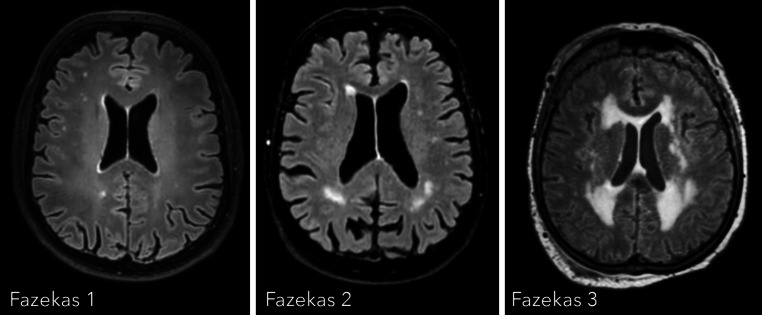
Fazekas scale

**Fig. 5:**
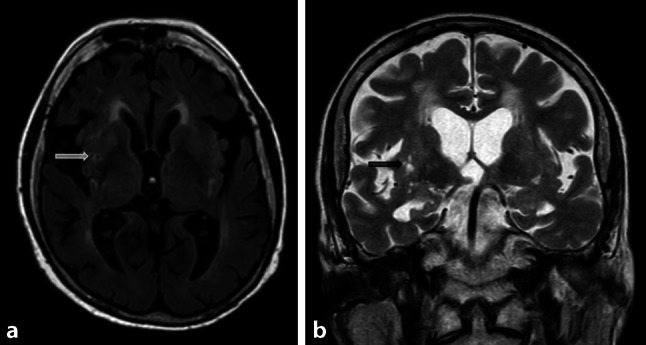
Lacunes. Illustration of lacunes in the putamen (*arrow*) on axial Flair images (**a**) and coronal T2-weighted images (**b**)

**Fig. 6:**
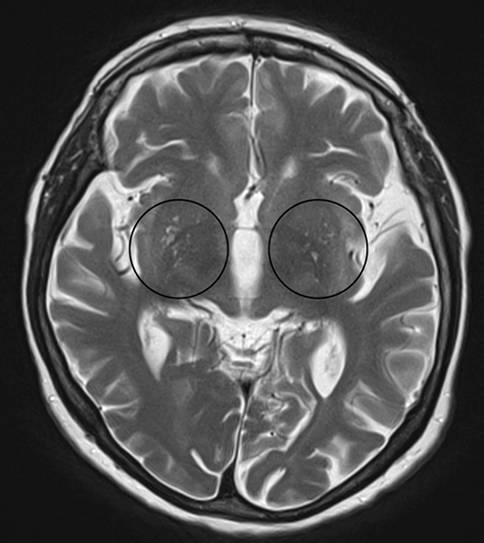
Virchow-Robin spaces. Illustration of enlarged Virchow-Robin spaces (*circles*) in the lower aspect of the basal ganglia on both sides on axial T2-weighted images

## Typical imaging features of the most common dementia subtypes

### Alzheimer’s disease

Neuroimaging is increasingly being incorporated as a biomarker in the diagnostic criteria for Alzheimer’s disease to increase diagnostic certainty [4]. The most common imaging finding of Alzheimer’s disease is cortical atrophy based on underlying neuronal loss. This atrophy predominantly affects symmetrically the parietal and temporal lobes. Late-onset Alzheimer’s disease and patients who present with APOE E4 polymorphism show, in particular, hippocampal atrophy (illustrated in Fig. 8), whereas the central region is relatively spared [5]. A significant asymmetric atrophy of the hippocampi does not exclude the diagnosis; however, this type of atrophy is more typical for alternative causes, such as frontotemporal dementia.

**Fig. 7:**
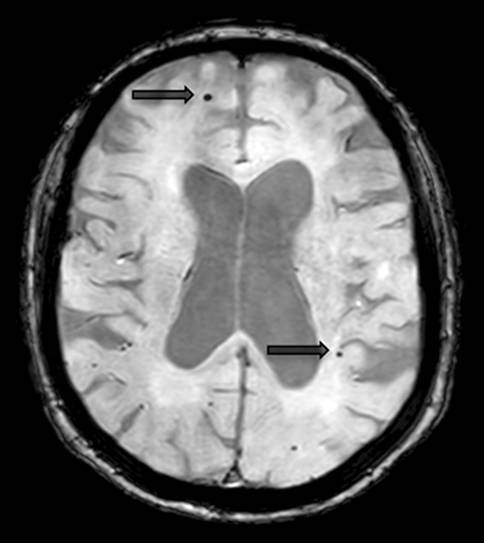
Microbleeds. Illustration of microbleeds on axial susceptibility-weighted images in the frontal and parietal lobe on both sides (the two most prominent microbleeds are marked with an *arrow*)

Patients with an atypical Alzheimer’s disease variant, such as early-onset Alzheimer’s disease or a missing APOE E4 genotype show only mild or no hippocampal atrophy, but, instead, present with considerable posterior cortical atrophy, e.g., the precuneus region in early-onset Alzheimer’s disease (illustrated in Fig. 9) [6]. In patients with posterior cortical atrophy, the parieto-occipital and the posterior temporal cortices are most commonly affected asymmetrically, more prominently on the right side, which results in an early visual or visuospatial impairment ahead of cognitive decline [7]. Patients with logopenic progressive aphasia show an asymmetric more prominent left-sided atrophy of the posterior temporal cortex and the inferior parietal lobe, which results in language-related impairments [8].

**Fig. 8:**
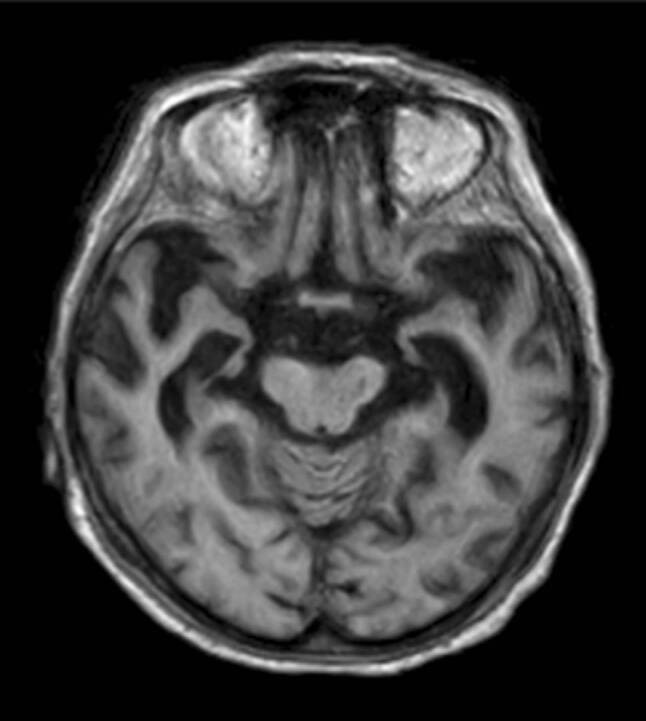
Alzheimer’s disease. A 67-old-patient with Alzheimer’s disease who presented with characteristic bilateral atrophy of the temporal lobe, including the hippocampi, depicted on axial T1-weighted images

### Vascular dementia

After Alzheimer’s disease, vascular dementia is the second most frequent type of dementia in the elderly. Vascular dementia summarizes various vascular pathologies, including multiple territorial ischemic infarcts, strategic ischemic infarcts (e.g., thalamic infarcts), multiple lacunar infarcts, Binswanger’s disease, cerebral autosomal dominant arteriopathy with subcortical infarcts, and leukoencephalopathy (CADASIL) or cerebral amyloid angiopathy. The difficulty with vascular dementia is the common overlap with changes in the normal aging brain or other diseases that cause dementia. Moreover, the frequent co-existence of Alzheimer’s disease and vascular disease raise suspicion about a direct causality between these two disease entities, often exacerbating pre-existing clinical or subclinical pathologies [9, 10].

Vascular dementia presents with small- and large-vessel disease mainly leading to confluent white matter changes, lacunar infarcts, and/or postischemic cortical/subcortical cerebrovascular lesions. Based on the NINDS-AIREN criteria, the diagnostic criteria for vascular dementia are as follows: a) confluent white matter changes involving  25% of the total white matter; b) lacunar infarcts involving the frontal white matter, multiple basal ganglia, as well as both thalami; or c) large-vessel infarcts involving bilateral anterior cerebral artery territories, the paramedian thalamic territory, the inferior medial temporal lobe, the parieto-temporal or temporo-occipital association areas, the angular gyrus, and the superior frontal and parietal watershed areas in the predominant hemisphere.

The imaging features are characterized by periventricular white matter hyperintensities, which can range from punctate to confluent (Fazekas 1–3), cortical/subcortical ischemic/postischemic lesions, lacunar infarcts, enlarged Virchow-Robin spaces, and/or microbleeds (see Fig. 10) [11, 12].

**Fig. 9:**
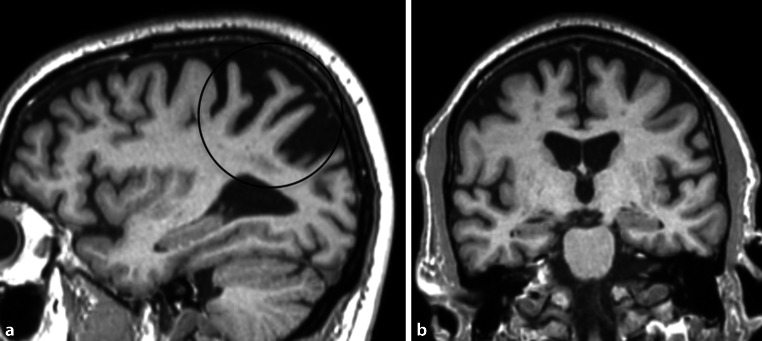
Atypical Alzheimer’s disease. A 58-year-old female patient with early-onset Alzheimer’s disease who presented with marked posterior cortical atrophy (depicted in **a** on sagittal T1-weighted images marked with a *circle*), while the hippocampi were relatively well preserved (shown in **b** on coronal T1-weighted images)

A particular subgroup of vascular disorders represents cerebral amyloid angiopathy, which results from amyloid deposits in the walls of blood vessels. Cerebral amyloid angiopathy is common in Alzheimer’s disease but is also found in the absence of Alzheimer’s disease neuropathological changes [13].

Neuroimaging is notable for diffuse, progressive white matter hyperintensities, microbleeds located in the cortical gray-white matter junction, superficial hemosiderosis, as well as acute intraparenchymal bleeding or post-hemorrhagic parenchymal defects (illustrated in Fig. 11) [14, 15].

**Fig. 10:**
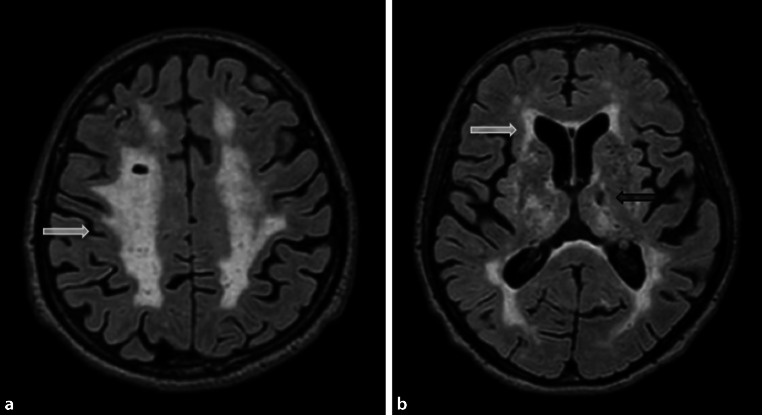
Vascular dementia. A 75-year-old patient presenting with extensive confluent periventricular white matter hyperintensities (*white arrow*, **a**, **b**), as well as a lacune in the left thalamus (*black arrow*) on axial Flair images

Another special subgroup of vascular disease is CADASIL, which is a hereditary vasculopathy based on mutations in the NOTCH3 gene [16, 17]. Patients with CADASIL show extensive white matter hyperintensities, lacunar infarctions, and hemorrhage mainly in the insulae and the anterior temporal lobes (illustrated in Fig. 12) [18].

**Fig. 11:**
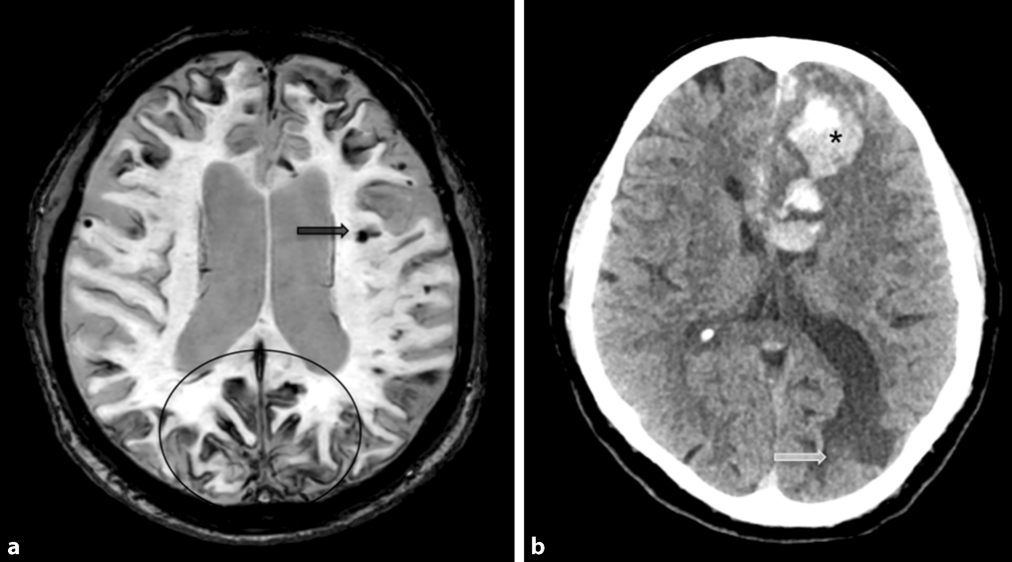
Cerebral amyloid angiopathy. A 60-year-old female patient with cerebral amyloid angiopathy who presented with superficial hemosiderosis, particularly frontal and occipital (the latter marked with a circle), as well as multiple microbleeds (*black arrow*) representing hemosiderin deposits on axial susceptibility-weighted images (**a**). There was also an acute intracranial bleeding in the left frontal lobe (*Asterisk*), as well as a post-hemorrhagic parenchymal defect in the left occipital lobe (*white arrow*) on axial computed tomography images (**b**)

### Frontotemporal dementia

Frontotemporal dementia consists of three different subtypes: the behavioral variant (40%); the progressive non-fluent aphasia (20%); and the semantic dementia (40%).

Patients who suffer from the behavioral variant of frontotemporal dementia show typically asymmetrical frontal and temporal cortical atrophy, with a gradient of the imaging findings from anterior to posterior, and a widening of the orbitofrontal sulci as one of the first signs of disease manifestation (see Fig. 13). Consequently, also the frontal horns of the lateral ventricles are widened disproportionately in the course of the disease. Atrophy also includes the insula, the anterior cingulate, the amygdala, the thalamus, and the striatum [19–21].

**Fig. 12:**
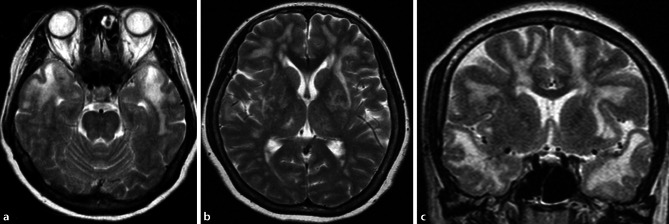
CADASIL. A 50-year-old female patient with CADASIL who presented with characteristic extensive white matter hyperintensities, particularly bilateral temporal, depicted on axial (**a**, **b**) and coronal (**c**) T2-weighted images

**Fig. 13:**
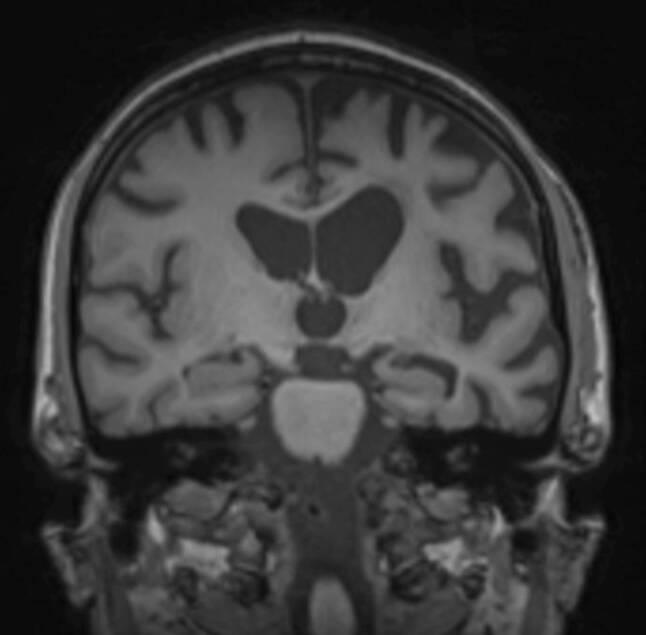
Frontotemporal dementia. A 60-year-old male patient with frontotemporal dementia, who presented with asymmetric frontotemporal atrophy depicted on coronal T1-weighted images

In patients with progressive non-fluent aphasia, the cortical atrophy is emphasized in the left-sided anterior perisylvian region, including the opercular and the insular regions as well as the premotor cortex, which results in effortful speech or agrammatic language production [22, 23].

In patients with semantic dementia, the cortical atrophy is particularly seen in the anterior and inferior temporal lobes (ventral and lateral regions), including the amygdala and the anterior hippocampus, again with a left-hemispheric predominance and an anterior-posterior gradient leading to an impairment of semantic performance, particularly impaired object knowledge and single-word comprehension [22, 23].

The logopenic progressive aphasia, the progressive non-fluent aphasia, and the semantic dementia can also be summarized as primary progressive aphasia.

### Dementia with Lewy bodies

Patients who suffer from dementia with Lewy bodies commonly show progressive cognitive impairments, especially in executive and visuospatial functions, followed by memory. The main clinical deviation compared to Alzheimer’s disease is, among others, the additional occurrence of visual hallucinations in patients who suffer from dementia with Lewy bodies. Moreover, on MR images the medial temporal lobes are relatively preserved in contrast to patients with Alzheimer’s disease [24].

## Conclusion

Dementia comprises various disease entities with different underlying pathologies. Neuroimaging can add additional information in the diagnostic work-up of patients who suffer from dementia. Therefore, the knowledge of typical imaging findings for the underlying causes of dementia is of essential value. Moreover, standardized imaging protocols, as well as structured radiological reports (including the use of predefined rating scales), will improve the diagnostic certainty and support interdisciplinary communication.
